# Disease stabilization with pembrolizumab for metastatic acral melanoma in the setting of autoimmune bullous pemphigoid

**DOI:** 10.1186/s40425-016-0123-3

**Published:** 2016-04-19

**Authors:** Kristen M. Beck, Joanna Dong, Larisa J. Geskin, Vincent P. Beltrani, Richard G. Phelps, Richard D. Carvajal, Gary Schwartz, Yvonne M. Saenger, Robyn D. Gartrell

**Affiliations:** Department of Medicine, Columbia University College of Physicians and Surgeons, New York, NY USA; Department of Dermatology, Columbia University College of Physicians and Surgeons, New York, NY USA; Department of Dermatology, Icahn School of Medicine at Mount Sinai, New York, NY USA; Department of Pathology, Icahn School of Medicine at Mount Sinai, New York, NY USA

**Keywords:** Pembrolizumab, Metastatic Melanoma, Bullous pemphigoid, Autoimmunity, Anti-PD1, Immunotherapy, Immune-related adverse events [irAEs]

## Abstract

**Background:**

To date, patients with pre-existing autoimmune conditions have been excluded from immunotherapy trials out of concern for severe autoimmune exacerbations.

**Case Presentation:**

We describe the first case of a patient with metastatic cKIT mutated acral melanoma, brain metastasis, and pre-existing severe autoimmune bullous pemphigoid (BP) with stable and asymptomatic disease 10 months after treatment with pembrolizumab. The patient experienced severe BP exacerbation after therapy with ipilimumab requiring systemic immune suppression, but nonetheless pembrolizumab was administered on further disease progression.

**Conclusions:**

This case suggests that pembrolizumab may confer more benefit than risk even in patients with known severe autoimmune conditions who require intermittent systemic immunosuppression.

## Background

Novel checkpoint blockade immunotherapies offer durable survival benefit for metastatic melanoma, although they remain of debatable benefit for patients who also have chronic autoimmune conditions because they confer significant risk of severe autoimmune toxicity. Incidences of autoimmune thyroiditis, hypophysitis, pneumonitis, hepatitis, nephritis, arthritis, tenosynovitis, and bullous pemphigoid (BP) after immune checkpoint blockade are well documented and have raised concern for use of immunotherapies in melanoma patients whose comorbidities predispose them to immune related adverse events (irAE) [1–5].

Current FDA-approved agents for unresectable metastatic melanoma include chemotherapy, BRAF inhibitors, MEK inhibitors, ipilimumab, a CTLA-4 inhibitor, and anti-PD1 antibodies pembolizumab and nivolumab. The current consensus among medical oncologists in the United States is that anti-PD1 agents should be included as first-line therapy, especially given the higher response rate and lower toxicity profile when compared to ipilimumab, and this is now reflected in National Comprehensive Cancer Network (NCCN) guidelines [1, 6, 7]. Checkpoint inhibition with anti-PD1 monoclonal antibodies increases overall survival in metastatic melanoma, and has demonstrated response in 25–40 % of patients [1, 8, 9]. In contrast, traditional chemotherapies (e.g., dacarbazine, temozolomide, fotemustine) have been of little efficacy for metastatic melanoma and are associated with a median survival of less than 9 months, with only a small minority (5–10 %) of patients achieving long-term survival of 5 years or more [9, 10]. Despite this, chemotherapy may be the only available therapeutic option for patients whose tumor does not express a readily targetable mutation and who cannot tolerate immunotherapy. Thus, chemotherapy may be recommended for patients with a history of severe autoimmune disease and metastatic melanoma since little is known about the safety and efficacy of PD-1 inhibitors in this population. Here, we report a case describing the effective use of a PD-1 inhibitor in stabilizing metastatic brain, lung, and bone lesions in a patient with a history of severe and concurrent BP and widely metastatic acral melanoma.

## Case presentation

A 72 year-old male was diagnosed with subungual melanoma removed from the nail bed of his right thumb in 2007 with a depth of 1.0 mm at the site of prior melanoma in situ dating back to 1981. In 2009, he had a recurrence at the same site extending down his digit and necessitating amputation of the distal phalanx. Resected sentinel nodes showed a melan-A positive focus of hyperchromatic atypical melanocytes in one node and S-100 positive dendritic cell staining in the other two nodes. Due to potential surgical complications and patient preference for observation, complete dissection was not performed.

**Fig. 1:**
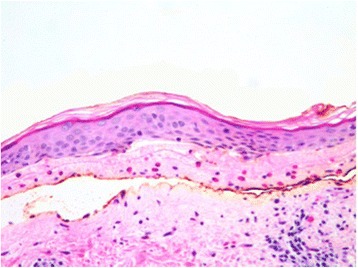
Biopsy of the patient’s skin lesions. Haematoxylin and eosin stain reveals subepidermal bulla as well as fibrin net, numerous eosinophils, perivascular mixed infiltrate, and well-preserved dermal papillae within the bulla cavity

In 2010, the patient presented with multiple bullae on the back. The diagnosis of BP was made by a skin biopsy and clinical and pathological correlation [Fig. 1]*.* Disease activity was managed with high dose oral prednisone with eventual taper and disease remission after one year. In 2011, the patient noticed a new lump in his right axilla. Pathology confirmed malignant melanoma with a cKIT mutation (L576P in exon 11) completely replacing a lymph node. Positron-emission tomography (PET) revealed lung nodules, suspicious for metastatic disease. The patient declined a recommended lung biopsy and, after discussion of the risk of BP flare, therapy was initiated with ipilimumab at 3 mg/kg. Several days after the second dose, he experienced severe exacerbation of his BP with mucous membrane involvement for which he was hospitalized and treated with a 9-week course of 60 mg of prednisone. He subsequently maintained control of BP with 15 mg of prednisone daily.

**Fig. 2:**
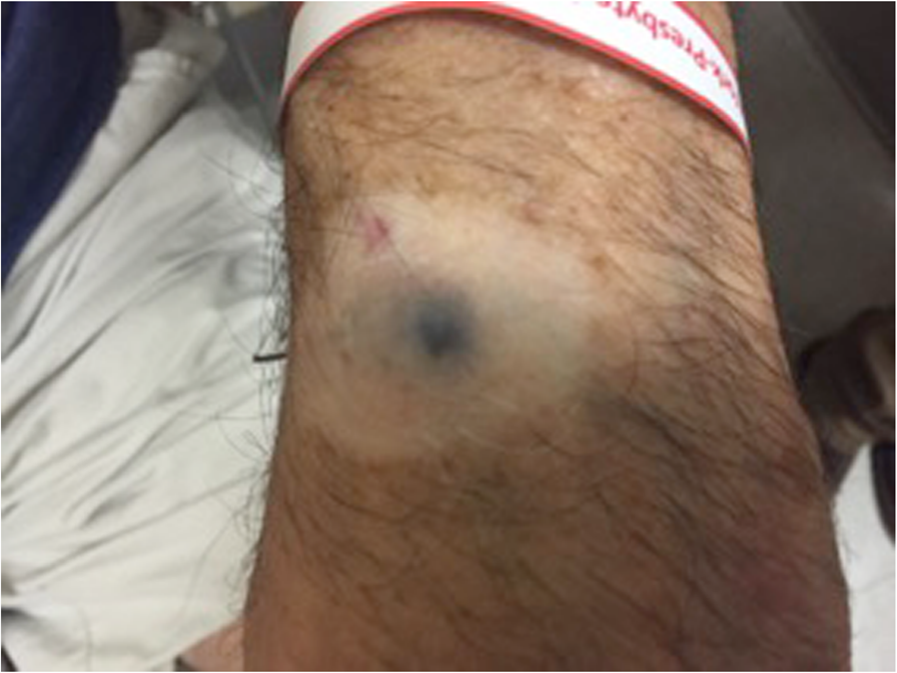
Cutaneous melanoma lesion with surrounding vitiligo

**Fig. 3:**
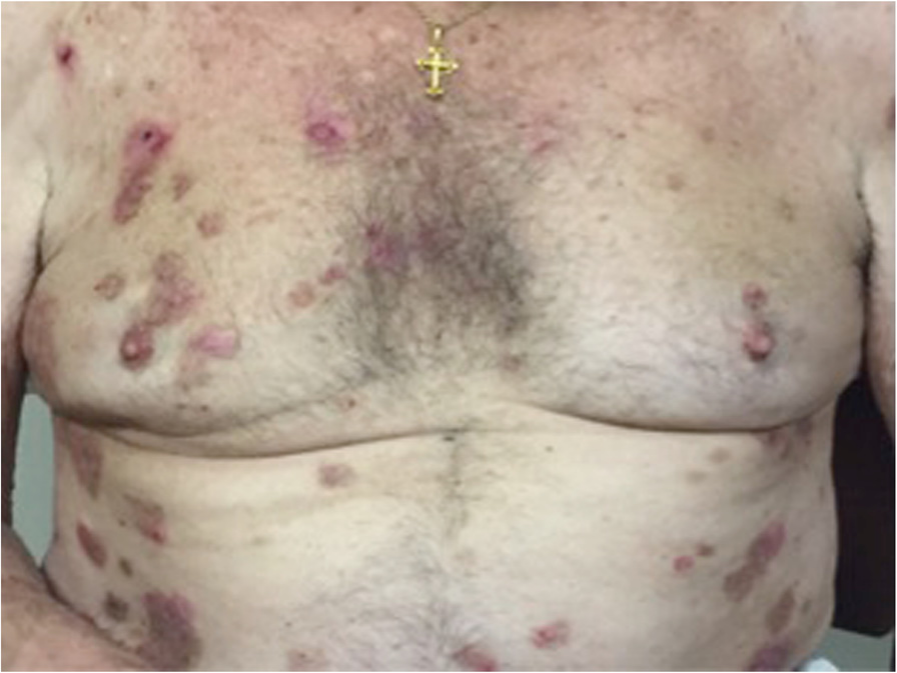
Clinical picture of ruptured bullae, erosions, and crusts of mild bullous pemphigoid exacerbation on low-dose corticosteroid treatment

After discontinuing ipilimumab, computed tomographic (CT) scan showed numerous enlarging bilateral pulmonary lesions and mediastinal lymphadenopathy. Magnetic resonance imaging (MRI) of the brain showed small volume brain metastases to the left internal auditory canal and the inferior aspect of right cerebellum, which were clinically asymptomatic. He declined radiation therapy for the brain lesion and began therapy with nilotinib, a small molecule tyrosine kinase inhibitor for cKIT mutant melanoma, at a dose of 400 mg twice daily. After three weeks of treatment, CT scan showed interval improvement in previously noted mediastinal lymphadenopathy. Over the following year, his nilotinib dose was reduced to 200 mg BID or held due to elevated liver function tests and prolonged QT interval. During this time, imaging showed interval progression of his brain and lung metastases. He received stereotactic radiosurgery to the right cerebellar lesion with subsequent stabilization of the brain lesions on imaging.

In 2014, he developed new osseous metastases to the thoracic vertebrae. Nilotinib was discontinued and he received palliative radiation to the spine for local disease control. At this time the patient completed prednisone taper without flare of his BP. The patient then received pembrolizumab, at a dose of 2 mg/kg given every three weeks. The first 3 doses of pembrolizumab were well tolerated with minimal bullous eruption, which was controlled with clobetasol 0.05 % topical cream. Imaging after 10 weeks of treatment showed essentially stable disease, and a cutaneous metastasis on his wrist flattened and regressed [Fig. 2]. However, following the fourth cycle, the patient developed a severe BP flare resulting in discontinuation of pembrolizumab and the start of oral prednisone at 60 mg a day. Over the next three months, the patient’s BP lesions had resolved and prednisone was tapered off, allowing for a fifth cycle of pembrolizumab [Fig. 3]. The patient again developed a flare of BP requiring patient to stop anti-PD1 and again start prednisone at the same dose. He had improvement of skin lesions but was unable to taper below 15 mg without worsening symptoms. One year following initial treatment with pembrolizumab, scans show stable disease with unchanged brain, pulmonary, and osseous metastases. Clinically, the patient feels well and denies symptoms related to his metastases. He is undergoing continued monitoring of both his metastatic melanoma and his BP.

## Discussion and conclusions

Patients with autoimmune disease have been excluded from checkpoint inhibitor trials out of concern for autoimmune exacerbation due to therapeutic blockade of checkpoint receptors. To our knowledge, this is the first description of a patient with pre-existing and active autoimmune disease treated with pembrolizumab resulting in disease stabilization. As expected, ipilimumab and pembrolizumab treatment induced autoimmune disease flares in the patient. While the flare from ipilimumab was severe enough to warrant extreme caution regarding further administration of this agent, subsequent administration of pembrolizumab with a limited dose scheduling resulted in clinically meaningful disease control while autoimmune symptoms were controlled with oral steroids when needed. Notably, even modified intermittent use of PD-1 inhibitor resulted in stabilization of his melanoma in the presence of numerous markers of poor prognosis.

BP is a subepidermal blistering disease characterized by an autoimmune response to hemidesmosomal proteins within the dermal-epidermal junction that occurs primarily in elderly adults. It presents with pruritic urticarial plaques and tense subepithelial blisters on the skin [11]. The pathophysiology is similar to that of other autoimmune diseases: breakdown of T and B-cell tolerance to BP antigens leads to autoantibody production and subsequent blister induction [12]. The initiating trigger for the autoimmune response is yet unclear, though certain medications or infections have been associated [13, 14]. Although data is conflicting, there is also an association between BP and malignancy, suggesting that BP may arise as a paraneoplastic condition [15]. In this case, the patient’s diagnosis of BP predated his cancer diagnosis, making the possibility of a paraneoplastic bullous pemphigoid less likely. There is one prior case report of a patient developing new-onset BP during pembrolizumab therapy, without resumption of further cycles after immunosuppressant therapy, and subsequent progression of brain metastasis [5]. In this case, BP exacerbations were clearly temporally associated with immunotherapy, but adequate control of BP in a multidisciplinary fashion allowed for continuation of potentially life-prolonging medication.

The major goal of checkpoint blockade with PD-1 and CTLA-4 is to interfere with the ability of tumors to stimulate inhibitory surface receptors on T cells and generate potent tumor immunity [16]. However, given that these receptors are critical to maintaining immunologic homeostasis, there is theoretically concern that these therapies pose significant risk to patients with pre-existing conditions of autoimmunity. The differences between CTLA-4 and PD-1 in both expression and mechanism of action are thought to contribute to the differences in autoimmune side effects of their respective therapeutic inhibitors. CTLA-4 is expressed by T-cells throughout the body, and its ligand is expressed by antigen presenting cells. CTLA-4 is also highly expressed on Foxp3+CD4+ regulatory T cells (Tregs) and there is ample evidence that CLTA-4 is a locus of susceptibility to autoimmune disease. In contrast, PD-1 expression is limited primarily to exhausted T-cells and its ligand is found on tumor cells. PD-1 is therefore more antigen-specific than CTLA-4. The therapeutic blockade of CTLA-4 on Tregs profoundly impairs the ability of Tregs to attenuate T cell activation required for peripheral tolerance. Therefore, PD-1 blockade is associated with milder autoimmune side effects, in comparison to the severe, nonspecific autoimmune side effects of CLTA-4 blockade. Also for these reasons, autoimmune flares on anti-CTLA-4 therapy do not necessarily recur with subsequent anti-PD1 therapy. A recent study of patients with irAE on ipilimumab exhibited unrelated or nonexistent irAEs when subsequently treated with pembrolizumab [17]. Our case is consistent with this report, as the patient experienced severe irAE requiring hospitalization after treatment with ipilimumab, but was able to tolerate pembrolizumab with milder irAE.

Nevertheless, dermatologic adverse events are among the most common irAEs in patients treated with all checkpoint inhibitors. The rash observed with checkpoint inhibitors is most often reticular, edematous, and maculopapular [18]. It may be asymptomatic or pruritic. Current guidelines for the management of irAEs generally recommend immunosuppression with corticosteroids as well interruption of the checkpoint inhibitor treatment cycle until symptoms or toxicity are grade 1 (mild) or less [19]. Grade 1 and 2 (mild and moderate) rashes can be treated with topical corticosteroids and oral antihistamines. Grade 1 and 2 oral mucositis can often be managed with oral corticosteroid rinses and lidocaine. For grade 3 or 4 (severe or life-threatening) dermatologic irAEs, the checkpoint inhibitor should be discontinued and systemic corticosteroids are recommended. Prompt referral to a dermatologist and biopsy is indicated for any rash that does not respond to topical corticosteroids or that presents with bullous lesions. The differential diagnosis should include Stevens-Johnson syndrome and toxic epidermal necrolysis, life-threatening emergencies which have been reported in rare cases.

The optimal scheduling of CTLA-4 and PD-1 inhibition for best survival rate and minimal irAEs in the setting of concurrent autoimmune disease is not yet known. While 2 mg/kg every 3 weeks is recommended for pembrolizumab, clinical trials have incorporated higher doses and more frequent scheduling, which may account for the high rate of irAE seen. It is possible that such irAEs are dose-dependent. The kinetics of immunotherapy are such that responses may develop after many weeks and months of therapy, with benefits lasting even after discontinuation [20]. Responses to immunotherapy may also develop after only a few treatments. There is a reported case of tumor eradication after only one treatment with combination anti-PD1 and anti-CTLA-4 immunotherapies [21]. Importantly, based a study of 576 melanoma patients pooled from nivolumab clinical trials, immunosuppressive treatment for irAEs does not appear to impact outcomes [22]. As in our patient who received less frequent dosing due to autoimmune exacerbation, pembrolizumab with a lower frequency of dosing is a reasonable strategy for patients with refractory metastatic melanoma and concurrent autoimmune disease. Thus, this case illustrates that dosing and or frequency of immunotherapy may need to be individualized based on autoimmune toxicity.

Pembrolizumab’s ability to halt progression of disease and promote longer-term survival is also notable in this case because of this patient’s numerous poor prognostic factors. Acral melanoma, an uncommon subtype of melanoma with a classically poor prognosis and more aggressive invasiveness, has exhibited encouraging responsiveness to immunotherapy [23, 24]. cKIT mutations have been implicated as an independent risk factor for poor prognosis, and patients with cKIT mutations have shown lower rates of survival compared to those without cKIT mutations [25]. Brain metastases in melanoma have an extremely poor prognosis, though there is increasing evidence on the efficacy of pembrolizumab [26].

It is of paramount importance to develop strategies for use of immunotherapy to improve the chances of achieving durable benefit and long-term survival in patients with autoimmune conditions and/or who require chronic immunosuppression. Our case advocates for pembrolizumab treatment for patients with melanoma and refractory brain metastases who have progressed on other therapies. Given the life-threatening nature of advanced melanoma and the potential survival benefit conferred by pembrolizumab, treatment with pembrolizumab may be a reasonable option in the setting of autoimmune conditions, with careful consideration of dosages and cycle frequency.

## Consent

Written informed consent was obtained from the patient for publication of this case report and any accompanying images. Copies of the written consents are available for review by the Editor-in-Chief of this journal.

## Abbreviations

BID: twice a day
BP: bullous pemphigoid
CT: computed tomographic
CTLA-4: cytotoxic T-lymphocyte-associated antigen-4
irAE: immune-related adverse event
Mg/kg: milligram per kilogram
MRI: magnetic resonance imaging
NCCN: national Comprehensive Cancer Network
PD-1: programmed cell death-1
PET: positron-emission tomography
Tregs: Foxp3+CD4+ regulatory T cells
